# Effects of Defoliation Treatments of Babica Grape Variety(*Vitis vinifera* L.) on Volatile Compounds Content in Wine

**DOI:** 10.3390/molecules27030714

**Published:** 2022-01-21

**Authors:** Toni Kujundžić, Vesna Rastija, Domagoj Šubarić, Vladimir Jukić, Florian Schwander, Mato Drenjančević

**Affiliations:** 1 Faculty of Agrobiotechnical Sciences Osijek, Josip Juraj Strossmayer University of Osijek, Vladimira Preloga 1, 31000 Osijek, Croatia; toni.kujundzic@fazos.hr (T.K.); domagoj.subaric@fazos.hr (D.Š.); vladimir.jukic@fazos.hr (V.J.); mato.drenjancevic@fazos.hr (M.D.); 2 Julius Kühn-Institut, Federal Research Centre of Cultivated Plants, Institute for Grapevine Breeding Geilweilerhof, 76833 Siebeldingen, Germany; florian.schwander@julius-kuehn.de

**Keywords:** defoliation, wine, volatile compounds, gas chromatography

## Abstract

The aim of this study was to determine the effects of defoliation performed in the Babica red grape variety on the volatile compounds in produced wine. Three treatments were performed during 2017 and 2018: the removal of six leaves before flowering (FL) and at the end of veraison (VER), as well as control (C). Volatile compounds were analyzed using a gas chromatograph coupled to a mass spectrophotometric detector. Results were statistically evaluated by analysis of variance (ANOVA at the *p* = 0.05 level) and principal component analysis (PCA). Defoliation treatments were affected by the concentration of several compounds, but only in one year. The VER2017 treatment significantly increased the concentration of three aliphatic esters up to 8 C atoms and octanoic acid ethyl ester. The FL2017 treatment increased the concentration of three aliphatic alcohols. The FL2018 treatment has significantly enhanced the concentration ethyl cinnamate but decreased the concentrations of eugenol and dihydro-2-methyl-3(2*H*)-thiophenone. Both defoliation treatments reduced the concentration of γ-decanolactone in 2017. Aldehydes, monoterpenoles, and monoterpenes remained unaffected by the defoliation treatments. Vintage was found to be the largest source of variability for most volatile compounds under investigation, which was confirmed by PCA. The effect of defoliation in the mild-Mediterranean climate was found to mostly depend on seasonal weather conditions.

## 1. Introduction

The aroma of the wine is created by volatile compounds comprising a wide range of organic compounds, such as esters, aldehydes, ketones, alcohols, terpenes, acids, and volatile phenols. Many of these compounds can be detected at a very low concentration (μg/L). Wine aroma compounds originate from the grape aroma, as well as compounds generated by the fermentation and aging of wine [1,2,3]. The volatile aroma compounds in grapes are derived from the oxidation of fatty acids through the lipoxygenase pathway. Although the levels of volatile compounds in ripe grapes depends on variety, they are greatly affected by seasonally specific climate conditions, sunlight, fungal infections, and vineyard practices that include water management, crop thinning, and defoliation [4,5,6,7].

Defoliation is an ampelotechnical practice that improves the photosynthetic capacity of grapes, increases canopy air circulation, and improves the cluster microclimate, which leads increased crop plant and wine quality [8,9]. A study by Verzera et al. [10] showed that early defoliation could improve wine quality in the Mediterranean region due to increased concentrations of acids, furfural aldehydes, and C13-norisoprenoids. Another study showed that defoliation increased the concentrations of thiols and linalool in South African Sauvignon Blanc wine [11]. Our previous study about defoliation treatments in Cabernet Sauvignon wines from the eastern continental part of Croatia showed that volatile organic acids and alcohols remained mainly unaffected but the concentrations of some esters was reduced [12].

The outcome of defoliation treatments on wine volatile profiles is associated with many factors. The timing of defoliation has a great influence on grape and wine quality. Early defoliation around flowering lowers carbohydrate supply, and the result is a lower fruit set and fruit yield [13]. Early leaf removal applied in warm climatic conditions induces increases in the concentrations of all volatile compounds except for lactones [14]. Leaf removal around veraison (berry ripening initiation) or “late defoliation” effects the synthesis of primary and secondary metabolites, especially polyphenols [15,16], but its influence on the production of different groups of volatile compounds is still unclear since the intensity of sunlight exposure has a distinct influences on the production of volatile compounds [17,18]. A study of the effects of the different defoliation treatments that changed the light environment at the fruit zone on the concentration of volatile compounds in Muscat grapes (*Vitis vinifera* L.) showed that more severe defoliation (60–80% fruit light exposure) resulted in high concentrations of volatile compounds, particularly monoterpenes [19]. The defoliation intensity of Nero di Troia grapes and vine position in the vineyard was found to result in different concentrations of volatile compounds in wines; the highest concentrations of total ethyl esters were found in wines made from the grapes with a full leaf removal along the west side of the vineyard (and along the east side in pre-harvest), while the lowest terpene concentrations were found in the wines of the leaf-removed grapes in the fruit zone along the east side of the vineyard [20].

Leaf removal treatments are generally performed in cool regions with moderate sunlight and rainfall conditions [12,21,22,23]. Leaf removal promotes sunlight exposure and airflow, as well as reductions in foliage cover and disease incidence. Ultraviolet-B (UV-B) solar radiation induces grape berries to produce volatile compounds that protect tissues from UV-B itself and other abiotic and biotic stresses [24].

Due to possible sunburn damage and losses of grape quality, leaf removal technique is avoided in dry hot climates [8,25]. However, some studies have demonstrated that early leaf removal positively influences fruity and floral aromas in warm climatic conditions, such for the Nero d’Avola [10], and Tempranillo wines [7,14]. Early leaf removal led to changes in the aroma profile of *Vitis vinifera* L. cv. Tempranillo wines, but inconsistent trends between the two seasons were also noted, showing a vintage effect [26]. In dry-hot seasons of the Chines Xinjiang region, leaf removal caused reductions in the levels of main monoterpenes, norisoprenoids, and C6-derivated esters [27]. Babica is an autochthonous red grape variety from the Croatian wine region Dalmatia, where the climate is mild-Mediterranean, with hotter summers and mild winters. This grape variety was spontaneously created by natural genetic mixing. The aim of this study was to determine the effects of defoliation in the Babica grape variety (*Vitis vinifera* L.) before flowering and at the end of veraison on the volatile compounds in produced wine.

## 2. Results and Discussion

Defoliation treatments on the Babica grape variety in the Kaštela–Trogir vineyards were performed during the vegetation periods of 2017 and 2018. The weather conditions in Kaštel Štafilić during the experiment are shown in Table 1.

Seven groups of volatile compounds in Babica wine were identified: aliphatic esters (up to C8); aliphatic esters (C9 and higher); aliphatic alcohols; aromatic esters and alcohols; ketones; and aldehydes, monoterpenoles, and monoterpenes. The effects of defoliation treatments and vintage on the concentration of individual volatile compounds (μg/L) are shown in Table 2 for the aliphatic esters (up to C8), Table 3 for the aliphatic esters (C9 and higher), Table 4 for the aliphatic alcohols, Table 5 for the aromatic esters and aromatic alcohols, Table 6 for the ketones, and Table 7 for the aldehydes, monoterpenoles, and monoterpenes.

The most abundant aliphatic esters up to eight carbon atoms were found to be 3-methyl-1-butanol-acetate (isoamyl acetate) (**9**), hexanoic acid ethyl ester (ethyl caproate) (**15**), and butanedioic acid diethyl ester (ethyl succinate) (**23**) (the average concentrations were 76.1201, 56.9375, and 85.5258 μg/L, respectively) (Table 2). Aliphatic esters are produced during the fermentation from alcohol and acyl-CoA by yeast alcohol acyltransferase enzymes, giving wine a sweet fruity aroma [1]. Defoliation treatments were influenced by the few aliphatic esters, but only in 2017. Accordingly, the removal of six leaves at the end of veraison (VER2017) significantly decreased the concentrations of 2-methylbutanoic acid-ethyl ester (**7**) and 3-methylbutanoic acid ethyl ester (**8**) but increased the concentrations of 2-hydroxy-propanoic acid-ethyl ester (**18**), 2-hydroxy-propanoic acid-2-methylpropyl ester (**19**), and 3-methylbutyl 2-hydroxypropanoate (**22**). Since the concentration of aliphatic esters of medium-chain fatty acids depends on the concentration of the fatty acid precursor in grapes, defoliation at the end of veraison was probably influenced by the increased production of propanoic acid in grapes in 2017, which was found to result in an increased concentration of the propanoic acid esters [2]. Similarly, leaf and lateral shoot removal, which caused the highest mean maximum hourly UV radiation, increased the concentration of fatty acid ethyl esters in Sauvignon Blanc wine from the southern coastal area of South Africa such as ethyl butyrate, ethyl hexanoate, and ethyl octanoate [11]. Additionally, apical defoliation was shown to lead to an increase or decrease in the concentrations of diethyl butanedioate, ethyl 2-methylbutanoate, and ethyl 3-methylbutanoate in 2011 and 2016, respectively [25]. Unlike the study of Moreno, et al. [14], where it was observed that basal defoliation increased the concentration of isoamyl acetate in wine, the present treatments did not reveal in a significant difference.

Different effects of season could be observed in the content of aliphatic esters up to eight carbon atoms. For example, in the case of the most abundant esters, wines from 2018 had significantly higher concentration of isoamyl acetate (**9**) but lower concentrations of hexanoic acid ethyl ester ethyl caproate (**15**), 2-hydroxy-propanoic acid-ethyl ester (**18**), and ethyl succinate (**23**).

The contents of the aliphatic esters with C9 and higher (Table 3) was generally unaffected by leaf removal treatments, except for octanoic acid ethyl ester (**30**), the concentrations of which were highest in 2017 due to defoliation at the end of veraison, as well as ethyl-9-decenoate (**33**) in the same year due to treatment before flowering. Similarly, the highest concentration of octanoic acid ethyl ester was observed in Tempranillo wines treated with mechanical defoliation at fruit set [7]. The removal of leaves before flowering was found to induce increases in the concentration of all esters of Tempranillo wines produced in warm climatic conditions [14], as well as ethyl esters in Nero d’Avola wines from Sicily [10].

The lower average daily temperature and lower cumulative rainfall during the vegetation period in 2017 (Table 1) resulted in significantly higher concentrations of pentanedioic acid diethyl ester (**34**) and octanoic acid ethyl ester (**30**) for those plants treated at the end of veraison. The most abundant aliphatic alcohols (Table 4) were found to be 2-methyl-1-butanol (**47**) and 3-methyl-1-butanol (isoamyl alcohol) (**48**), with average concentrations of 175.58 and 295.03 μg/L, respectively. Concentrations of aliphatic alcohols were generally unaffected by defoliation treatments, except for 3-pentanol (**54**) and 1-heptanol (**64**), the lowest concentration of which was observed in FL2017 wine, while the same treatment significantly increased the concentrations of *E*-2-hexen-1-ol (**59**), 2-ethyl-1-hexanol (**66**), and (*S*)-(+)-6-methyl-1-octanol (**72**). A similar effect of defoliation on higher alcohols could be observed in the part of Croatia with a moderate continental climate. In Sauvignon Blanc and Riesling wines from the Prigorje-Bilogora subregion [21], as well as in Cabernet Sauvignon wines from eastern Croatia [12], no difference between control and basal leaf removal treatments was observed. In contrast, the removal of all leaves below the clusters at the beginning of flowering led to higher concentrations of 3-methyl-1-butanol and 2-methyl-1-propanol in Merlot wines from Brazil [28]. An increase in C6 alcohols in defoliated wine was observed in Tempranillo wines from the north of Spain [27] and Tempranillo wines from the west of Spain [14], though only in one year. Additionally, basal leaf removal before flowering led to higher contents of aliphatic alcohols in Istrian Malvasia wines [13]. In contrast, apical defoliation resulted in much lower concentrations of 1-hexanol in Shiraz wines from Australia [25]. Vintage was found to be the main source of variability for most of the aliphatic alcohols, so higher concentrations were found in studied wines from 2017, probably due to weather conditions during ripening process (lower average daily temperature during vegetation period and lower cumulative rainfall; see Table 1). Specifically, the relationship between least irrigated treatments and greater alcoholic compound (2-methyl-1-propanol, 1-butanol, 3-methyl-1-butanol, and 1-pentanolin wine) contents was determined [29].

Within the group of aromatic esters and aromatic alcohols, those with the highest concentrations were phenylethyl acetate (**84**) (average: 576.27 μg/L) and 4-hydroxy-3-methoxy-benzoic acid ethyl ester (ethyl vanillate) (**97**) (average: 104.94 μg/L) (Table 5). Defoliation treatments only affected two compounds in 2018. The removal of leaves before flowering significantly enhanced the concentration of 3-phenyl-2-propenoic acid ethyl ester (ethyl cinnamate) (**88**) but decreased the concentration of eugenol (**90**). In Tempranillo wines, 2-phenylethyl acetate showed higher levels after early defoliation treatments compared to control wines [7]. Different effects of the year on the concentration of aromatic compounds were observed here. For example, the weather conditions in 2017 were much more favorable for benzyl alcohol (**85**) and ethyl vanillate (**97**), while higher concentrations of phenylethyl acetate (**84**) and 2,3-dihydro-benzofuran (**96**) were found in wines from 2018.

Ketones and aldehydes are minor groups of compounds present in wine that remain after fermentation [1]. The most represented ketones determined in Babica wine were 2(3*H*)-furanone, dihydro-5-pentyl (**113**) (cocos aldehyde) and *γ*-decanolactone (**114**), with average concentrations 109.51 and 46.21 μg/L, respectively (Table 6). The concentrations of all aldehydes were lower than 1 μg/L. Only two ketones were affected by the defoliation treatments. Both defoliation treatments significantly reduced the content of γ-decanolactone (**114**) in 2017 and dihydro-2-methyl-3(2*H*)-thiophenone (**107**) in 2018. Aldehydes, monoterpenoles, and monoterpenes were not affected by defoliation treatments (Table 7). In wines Nero d’Avola, there were significantly higher amounts of furfural aldehydes and C13-norisoprenoids than in controls [10]. Leaf removal increases monoterpenes and other volatile terpenes in ‘Sauvignon Blanc’, ‘Gewürztraminer’, and ‘Chardonnay Musqué’ grapes from subtropical highland climates [30]. Vintage was found to be the major source of the variability of the ketones, so the four of them had higher concentrations in 2017 (**100**, **101**, and **109**) while and three were more abundant in 2018 (**107**, **108**, and **116**). The concentrations of aldehydes, monoterpenoles, and monoterpenes were mostly not influenced by vintage.

**Figure 1:**
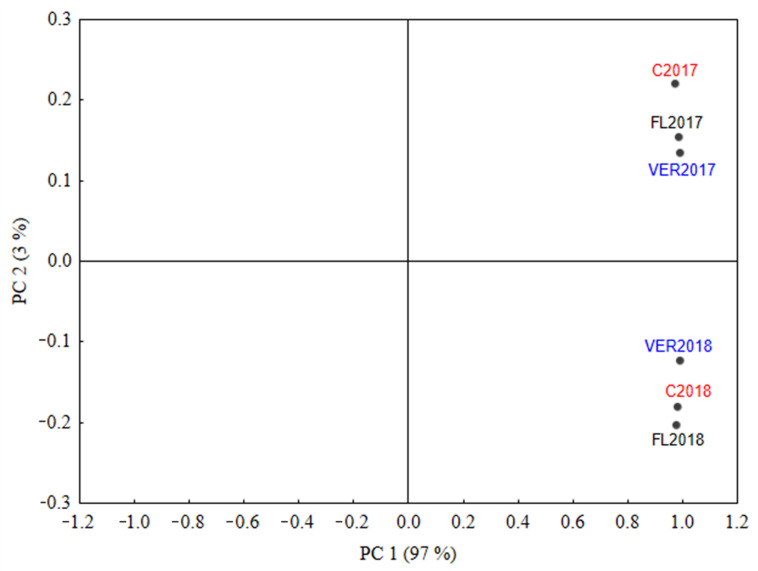
Two dimensional principal component analysis plot for the volatile compound concentrations in Babica wine made from control and two defoliation treatments in 2017 and 2018; distribution of the treatment year variable (C = control; FL = removal of six leaves before flowering; VER = removal of six leaves at the end of veraison).

Principal components analysis (PCA) was applied to evaluate the overall effect of the leaf removal treatments on the volatile compounds during the two years of study. Figure 1 shows a PCA plot for the two principal components in which the code for each point in the plot corresponds to the treatment year. PC1 explained 96.88% of the variation in the data, and all the treatment year variables were located on the positive side. PC2 explained 2.99% of the variation and separated the treatments by year. Wines from 2018 were located on the negative side of PC2, while wines from 2017 were located on its positive side. This confirmed the results of ANOVA analysis: the main source of variability of volatile compound concentrations was the vintage.

**Figure 2:**
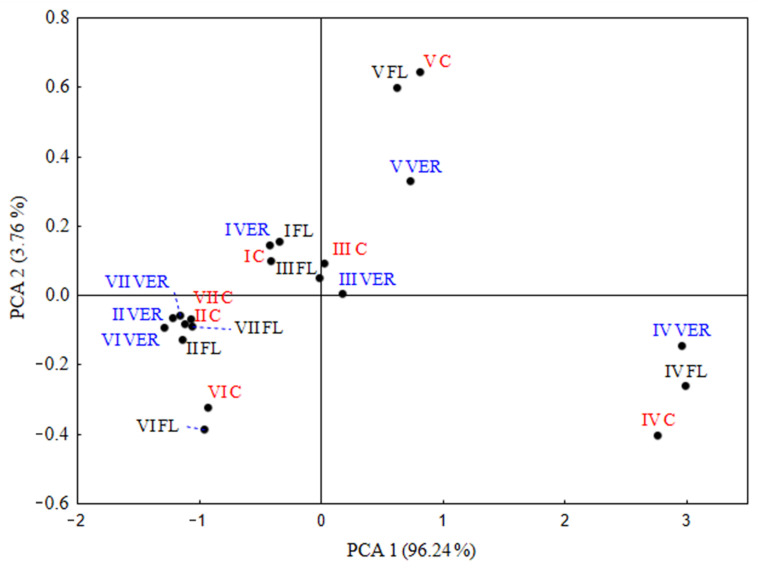
Two dimensional principal component analysis plot for average concentrations of the seven groups of volatile compounds (I: aliphatic esters (up to C8); II: aliphatic esters (C9 and higher); III: aliphatic alcohols; IV: aromatic esters and alcohols; V: ketones; VI: aldehydes; VII: Monoterpenoles and monoterpenes) determined in wine made from control and two defoliation treatments (C = control; FL = removal of six leaves before flowering; VER = removal of six leaves at the end of veraison).

Figure 2 shows the distribution of the average concentrations of the seven groups of volatile compounds (I: aliphatic esters (up to C8); II: aliphatic esters (C9 and higher); III: aliphatic alcohols; IV: aromatic esters and alcohols; V: ketones; VI: aldehydes; and VII: monoterpenoles and monoterpenes) determined in wine made from the control and two defoliation treatments. The first component explained 96.24% of the variation. The wines were most affected by defoliation treatments in 2018, where aromatic esters and alcohols (IV) were separately positioned on the positive lower part of PC1. Three groups of compounds (VII: monoterpenoles and monoterpenes; VI: aldehydes; and II. aliphatic esters (C9 and higher)) that remained unaffected by any treatments in 2017 were positioned on the negative side of PC1 and the negative side of PC2. The effect of leaf removal in 2017 was significant for the aliphatic esters (up to C8) that were positioned on the negative sides of PC1 and PC2. aliphatic alcohols from the C and FL wines were very closely positioned on the zero values of PC1, while those from the VER wine were slightly separated at the positive side of PC1 and had zero value for PC2. Ketones (group V) from three different treatments were singled out in the above positive part of PC1.

## 3. Materials and Methods

### 3.1. Plant Material and Experimental Design

This study was carried out with the Babica *Vitis vinifera* grape variety (VIVC variety number 23110) during 2017 and 2018. In vineyards in Kaštel Novi and Kaštela–Trogir of the Central and Southern Dalmatia subregion from the wine-growing region of Dalmatia, Croatia (43°33′12.4″ N 16°18′41.3″ E), grafted SO4 rootstock was planted in 2003 in a north–south orientation with a 1.6 spacing between the rows and a 1.0 m spacing between vines. The experiment was set up according to a random block design with three treatments in four replications (control treatment without leaf removal, removal of six leaves before flowering, and removal of six leaves at the end of veraison). The training system was low cordon (8 buds per vine) with a 50 cm trunk height. The treatments consisted of 7 plants per repetition, which comprised 84 vines in total. The experimental field provided no irrigation system. The soil management practices were all mechanically performed, and standard cultural practices in the Adriatic Croatia area were applied to all of the treatments.

### 3.2. Microvinification

From each replicate, grapes were destemmed, crushed, treated with 50 mg/L of SO_2_ before being inoculated with *Saccharomyces cerevisiae* (Lalvin D254) (30 g/hL). Fermentation was conducted in 5-L glass fermenters at a temperature of 25 °C. Pomace was mixed twice a day. After five days of fermentation and maceration, pomace was pressed using a small mechanical press. Wines were sulfited with 20 mg/L of SO_2_. Three months after the end of fermentation, wines were bottled and stored at 12 °C until required for analysis.

### 3.3. GS/MS Analysis of Volatile Compounds

#### 3.3.1. Preparation of Samples

A sample of wine (2 mL) was suspended in 20 mL of distilled water with 25% NaCl (*w*/*v*) in a glass vial. Stir bar sorptive extraction (SBSE) was performed using magnetic stir-bars with polydimethylsiloxane phase (film thickness of 1.0 mm and length of 10 mm) (Gerstel Twister^®^, Mühlheim an der Ruhr, Germany), which were reconditioned in an acetonitrile–methanol suspension (80:20, *v*/*v*) and heated in the tube conditioner (up to 300 °C for 230 min) under helium flow. Extraction was performed at 1000 rpm for 20 h.

#### 3.3.2. Chromatography

Volatile compounds were analyzed using Agilent 6890N gas chromatograph (GC) coupled to an Agilent 5975B mass spectrophotometric (MS) detector (Santa Clara, CA, USA). The GC was equipped with a thermal desorption unit (TDS), a cold injection system (CIS), and a multipurpose sampler (MPS2) (Gerstel, Mühlheim an der Ruhr, Germany). The capillary column was an HP-INNOWax polyethylene-glycol (Agilent, Santa Clara, CA, USA) 60 m × 320 μm × 0.25 μm). The carrier gas helium was maintained at a 1 mL/min flow. The initial GC oven temperature was 40 °C for 5 min, and it was then ramped up to 250 °C at 2 °C/min and held at that temperature for 30 min.

The identification of the separated compounds was performed with retention indices and MS spectra compared with the Wiley 7 Nist 05 mass spectral database. The quantification was conducted according to [31] using a linear calibration with the following structurally related standards (functional group and chemical structure) in ten calibration levels covering the concentration range of samples: 2-phenylethanol (for aromatic esters and aromatic alcohols), 1-hexanol (for aliphatic alcohols), acetic acid hexyl ester (for aliphatic esters up to C8), decanoic acid ethyl ester (for aliphatic esters C9 and higher), *α*-pinene and linalool (for monoterpenes and aldehydes), and *β*-damascenone (for ketones) [27]. System software control, data management, and analysis were performed with enhanced ChemStation Software (Agilent Technologies, Inc., Santa Clara, CA, USA). Concentrations of volatiles are expressed in equivalents of the reference compounds (µg/L). All samples were analyzed in triplicate. Presented data are the means of four repetitions in vineyards and three chromatographic measurements.

### 3.4. Statistical Analysis

Comparisons of volatile compound concentrations between different treatments and years were made using analysis of variance (one-way ANOVA) with the least significant different (LSD) test used to examine means at the *p* = 0.05 level. Principal component analysis was used to examine the effects of early leaf removal treatments on volatile compounds. Statistical analyses were performed with Statistica 14 (TIBCO Software Inc., 2020, Palo Alto, CA, USA).

## 4. Conclusions

Volatile compounds in the Babica wine variety from the mild-Mediterranean region of Dalmatia in Croatia remained mainly unaffected by defoliation treatments. Several esters showed increased concentrations following the removal of leaves at the end of veraison, though only in 2017. The removal of six leaves before flowering in 2017 significantly increased the concentrations of three aliphatic alcohols and decreased the concentration of 3-pentanol. Rarely observed effects of defoliation on aromatic alcohols, aromatic esters, and ketones were mostly observed in 2018, when the removal of leaves before flowering had significantly enhanced the concentration of ethyl cinnamate but decreased the concentration of eugenol. The removal of six leaves before flowering decreased the content of 3-hydroxy-2-butanone and dihydro-2-methyl-3(2*H*)-thiopentone in wine from 2018, and the highest concentration of *γ*-decanolactone was observed in control wine from 2017. Aldehydes, monoterpenoles, and monoterpenes remained unaffected by defoliation treatments. We can conclude that effects of defoliation in mild-Mediterranean climate mostly depend on seasonal weather conditions. Vintage was found to be the largest source of variability for most volatile compounds under investigation, as confirmed by principal component analysis.

## Figures and Tables

**Table 1:** Weather conditions in Kaštel Štafilić during vegetation periods in 2017 and 2018.

	Mean Daily Temperature, °C		Rainfall, mm	
Month/Year	2017	2018	2017	2018
April	13.7	17.1	56.2	62.1
May	19.5	21.3	42.5	78.8
June	25.1	24.2	4.6	63.3
July	26.9	26.4	4.4	32.2
Aug	28.2	27.5	1.4	36.1
Sept (until the harvest)	21.8	24.1	116.6	17.5
Cumulative rainfall, mm			225.7	290.0
Mean temperature, °C	22.5	23.4		

**Table 2:** Effects of defoliation treatments and vintage on concentrations of aliphatic esters (up to C8) (μg/L). (The quantification was conducted by using a linear standard calibration with acetic acid hexyl ester.) C = control; FL = removal of six leaves before flowering; VER = removal of six leaves at the end of veraison.

Code	CAS	Name	C2017	FL2017	VER2017	C2018	FL2018	VER2018
**1**	79-20-9	Methyl acetate	0.28 ± 0.06	0.22 ± 0.06 b	0.26 ± 0.09	0.37 ± 0.07	0.36 ± 0.01 a	0.34 ± 0.04
**2**	105-37-3	Propanoic acid ethyl ester	2.55 ± 1.96	3.17 ± 3.27	1.71 ± 0.76	1.23 ± 0.12	1.17 ± 0.06	1.11 ± 0.08
**3**	97-62-1	2-Methyl propanoic acid ethyl ester	1.93 ± 0.58	1.95 ± 1.35	1.28 ± 0.67	1.55 ± 0.21	1.52 ± 0.22	1.43 ± 0.05
**4**	109-60-4	Acetic acid propyl ester	0.33 ± 0.08 a	0.30 ± 0.13 a	0.27 ± 0.08 a	0.23 ± 0.03 b	0.20 ± 0.03 b	0.18 ± 0.02 b
**5**	110-19-0	Acetic acid-2-methylbutylester	1.13 ± 0.21 b	1.17 ± 0.13 b	1.01 ± 0.37 b	2.96 ± 0.53 a	3.11 ± 0.51 a	2.79 ± 0.44 a
**6**	105-54-4	Butanoic acid ethyl ester	2.48 ± 0.37 a	2.78 ± 0.67 a	2.47 ± 0.16 a	1.64 ± 0.14 b	1.86 ± 0.14 b	1.51 ± 0.28 b
**7**	7452-79-1	2-Methylbutanoic acid ethyl ester	2.31 ± 1.06 A	1.61 ± 0.72 AB	1.51 ± 0.61 B	1.40 ± 0.23	1.35 ± 0.29	1.25 ± 0.15
**8**	108-64-5	3-Methylbutanoic acid ethyl ester	1.57 ± 0.45 AB	1.97 ± 1.14 A	1.04 ± 0.26 B	1.46 ± 0.26	1.44 ± 0.09	1.33 ± 0.15
**9**	123-92-2	3-Methyl-1-butanol-acetat (isoamyl acetate)	40.24 ± 1.69 b	34.53 ± 11.44 b	32.02 ± 14.74 b	123.56 ± 30.76 a	127.86 ± 29.15 a	98.51 ± 26.3 a
**10**	539-82-2	Pentanoic acid ethyl ester	0.30 ± 0.07	0.31 ± 0.24	0.19 ± 0.01	0.18 ± 0.02	0.18 ± 0.02	0.16 ± 0.03
**11**	623-70-1	*E*-2-Butenoic acid ethyl ester	0.54 ± 0.19 a	0.49 ± 0.22 a	0.54 ± 0.15 a	0.19 ± 0.04 b	0.14 ± 0.02 b	0.14 ± 0.02 b
**12**	106-70-7	Hexanoic acid methyl ester	0.14 ± 0.04 a	0.13 ± 0.02 a	0.14 ± 0.01 a	0.08 ± 0.01 b	0.07 ± 0.0 b	0.08 ± 0.01 b
**13**	624-54-4	Propanoic acid pentyl ester	0.69 ± 0.12	0.35 ± 0.11	0.38 ± 0.09	0.36 ± 0.06	0.36 ± 0.04	0.34 ± 0.15
**14**	25415-67-2	4-Methyl-pentanoic acid ethyl ester	0.04 ± 0.01	0.05 ± 0.01	0.04 ± 0.01	0.04 ± 0.00	0.04 ± 0.00	0.03 ± 0.01
**15**	123-66-0	Hexanoic acid ethyl ester	71.03 ± 16.56 a	73.06 ± 6.46 a	76.27 ± 5.16 a	43.12 ± 2.15 b	40.28 ± 4.06 b	37.87 ± 3.12 b
**16**	54653-25-7	5-Hexenoic acid ethyl ester	0.11 ± 0.06	0.16 ± 0.02	0.03 ± 0.01	0.04 ± 0.00	0.04 ± 0.00	0.04 ± 0.01
**17**	2396-83-0	3-Hexenoic acid ethyl ester	0.01 ± 0.00	0.01 ± 0.00	0.01 ± 0.00	0.01 ± 0.01	0.01 ± 0.00	0.01 ± 0.00
**18**	97-64-3	2-Hydroxy-propanoic acid ethyl ester	11.27 ± 3.87 Ba	14.54 ± 4.37 Ba	24.9 ± 9.36 Aa	4.00 ± 2.61 b	4.18 ± 2.61 b	3.14 ± 2.09 b
**19**	186817-74-3	2-Hydroxy-propanoic acid-2-methylpropylester	0.01 ± 0.00 B	0.02 ± 0.00 Ba	0.03 ± 0.01 A	nd	0.01 ± 0.0 b	nd
**20**	10348-47-7	2-Hydroxy-pentanoic acid-4-methyl ethyl ester	1.17 ± 0.16	1.52 ± 0.85	1.16 ± 0.47	1.13 ± 0.38	0.87 ± 0.16	0.99 ± 0.16
**21**	13327-56-5	Ethyl-3-methylthio-propanoate	0.08 ± 0.07 b	0.09 ± 0.03 b	0.12 ± 0.04 b	0.39 ± 0.03 a	0.36 ± 0.04 a	0.38 ± 0.03 a
**22**	19329-89-6	3-Methylbutyl 2-hydroxypropanoate (isoamyl lactate)	0.29 ± 0.09 B	0.37 ± 0.15 Ba	0.59 ± 0.18 Aa	0.15 ± 0.06	0.16 ± 0.1 b	0.11 ± 0.09 b
**23**	123-25-1	Butanedioic acid diethyl ester (ethyl succinate)	115.95 ± 27.64 a	155.28 ± 73.59 a	114.21 ± 23.86 a	47.45 ± 13.30 b	41.70 ± 6.58 b	38.56 ± 4.57 b
**24**	999-10-0	4-Ethyl-hydroxybutanoate	0.27 ± 0.06 b	0.34 ± 0.05 b	0.29 ± 0.06 b	0.62 ± 0.09 a	0.72 ± 0.12 a	0.63 ± 0.06 a
**25**	2305-25-1	3-Hydroxy-hexanoic acid ethyl ester	5.20 ± 2.89	6.19 ± 6.83	4.34 ± 1.43	2.09 ± 0.11	1.80 ± 0.17	1.89 ± 0.20

These data are from the means of four values ± standard deviation (SD). Different upper case letters in each row indicate statistically significant differences (*p* < 0.05) between treatments in the same year, as determined with the Fisher’s least significant different (LSD) test. Different lower case letters in each row indicate statistically significant differences (*p* < 0.05) between years for the same treatment, as determined with Fisher’s test. nd = not detected.

**Table 3:** Effects of defoliation treatments and vintage on concentrations of aliphatic esters (C9 and higher) (μg/L). (The quantification was conducted by using a linear standard calibration with decanoic acid ethyl ester.) C = control; FL = removal of six leaves before flowering; VER = removal of six leaves at the end of veraison.

Code	CAS	Name	C2017	FL2017	VER2017	C2018	FL2018	VER2018
**26**	106-27-4	Butanoic acid-3-methylbutyl ester	0.05 ± 0.01	0.07 ± 0.01	0.04 ± 0.00	0.02 ± 0.00	0.03 ± 0.01	0.05 ± 0.01
**27**	626-77-7	Propyl-hexanoate	0.01 ± 0.00	0.01 ± 0.00	0.01 ± 0.00	nd	nd	nd
**28**	106-30-9	Heptanoic acid ethyl ester	0.03 ± 0.01	0.04 ± 0.02	0.04 ± 0.01	0.02 ± 0.00	0.02 ± 0.00	0.02 ± 0.00
**29**	111-11-5	Octanoic acid methyl ester	0.05 ± 0.03	0.06 ± 0.01	0.07 ± 0.01	0.05 ± 0.01	0.05 ± 0.01	0.05 ± 0.01
**30**	106-32-1	Octanoic acid ethyl ester	9.85 ± 6.28 B	12.01 ± 1.07 AB	15.65 ± 1.38 Aa	9.96 ± 3.85	8.35 ± 3.21	8.53 ± 2.34 b
**31**	35194-38-8	7-Octenoic acid ethyl ester	0.01 ± 0.00	0.01 ± 0.00	nd	0.01 ± 0.00	nd	nd
**32**	123-29-5	Nonanoic acid ethyl ester	0.11 ± 0.00	0.10 ± 0.00	0.11 ± 0.00	0.13 ± 0.10	0.07 ± 0.01	0.16 ± 0.01
**33**	67233-91-4	Ethyl-9-decenoate	0.03 ± 0.01 B	0.08 ± 0.03 A	0.06 ± 0.01 AB	0.07 ± 0.03	0.09 ± 0.04	0.07 ± 0.04
**34**	818-38-2	Pentanedioic acid diethyl ester	0.06 ± 0.01 a	0.06 ± 0.03 a	0.05 ± 0.02 a	0.03 ± 0.01 b	0.02 ± 0.00 b	0.02 ± 0.00 b
**35**	67233-92-5	Succinic acid butyl ethyl ester	0.94 ± 0.31	1.10 ± 0.47 b	0.80 ± 0.23	0.63 ± 0.21	0.56 ± 0.10 a	0.56 ± 0.04
**36**	106-33-2	Dodecanoic acid ethyl ester	0.04 ± 0.01	0.05 ± 0.03 a	0.04 ± 0.01	0.02 ± 0.00	0.02 ± 0.0 b	0.02 ± 0.00
**37**	74367-34-3	Propanoic acid, 2-methyl-, 3-hydroxy-2,4,4-trimethylpentyl ester	0.75 ± 1.41	0.08 ± 0.06	0.05 ± 0.00	0.06 ± 0.01	0.06 ± 0.01	0.06 ± 0.01
**38**	74367-33-2	Propanoic acid, 2-methyl-, 2,2-dimethyl-1-(2-hydroxy-1-methylethyl) propyl ester	0.82 ± 1.53	0.10 ± 0.09	0.06 ± 0.00	0.07 ± 0.01	0.07 ± 0.00	0.07 ± 0.00
**39**	28024-16-0	Ethyl-3-methylbutyl-butanedienoate	1.81 ± 0.86	2.18 ± 1.17	1.55 ± 0.53	1.63 ± 0.67	1.37 ± 0.23	1.26 ± 0.13
**40**	107141-15-1	3-Hydroxy-tridecanoic acid ethyl ester	1.03 ± 0.03	1.23 ± 0.04	0.83 ± 0.04	0.59 ± 0.09	0.58 ± 0.09	0.49 ± 0.10
**41**	142-91-6	Isopropyl palmitate	0.24 ± 0.02	0.18 ± 0.02	0.24 ± 0.02	0.10 ± 0.01	0.06 ± 0.00	0.09 ± 0.00
**42**	105-99-7	Hexanedioic acid, dibutyl ester	0.06 ± 0.00	0.06 ± 0.00	0.06 ± 0.00	0.07 ± 0.00	0.07 ± 0.00	0.07 ± 0.00
**43**	24851-98-7	Methyl-dihydrojasmonate	0.30 ± 0.01	0.28 ± 0.01	0.32 ± 0.08	0.30 ± 0.01	0.36 ± 0.01	0.50 ± 0.02
**44**	183613-15-2	3-Hydroxy-dodecanoic acid ethyl ester	0.04 ± 0.01	0.05 ± 0.01	0.06 ± 0.01	0.01 ± 0.00	0.01 ± 0.00	0.01 ± 0.00

These data are from the means of four values ± standard deviation (SD). Different upper case letters in each row indicate statistically significant differences (*p* < 0.05) between treatments in the same year, as determined with Fisher’s least significant different (LSD) test. Different lower case letters in each row indicate statistically significant differences (*p* < 0.05) between years for the same treatment, as determined with Fisher’s test. nd = not detected.

**Table 4:** Effects of defoliation treatments and vintage on concentrations of aliphatic alcohols (μg/L). (The quantification was conducted by using a linear standard calibration with 1-hexanol.) C = control; FL = removal of six leaves before flowering; VER = removal of six leaves at the end of veraison.

Code	CAS	Name	C2017	FL2017	VER2017	C2018	FL2018	VER2018
**45**	78-83-1	Isobutanol	38.19 ± 13.79 b	36.83 ± 5.52 b	39.29 ± 1.91 b	53.47 ± 9.49 a	51.43 ± 3.63 a	59.28 ± 7.1 a
**46**	71-36-3	1-Butanol	1.33 ± 1.02	1.61 ± 1.55	2.19 ± 1.90	0.77 ± 0.07	0.79 ± 0.07	0.67 ± 0.09
**47**	137-32-6	2-Methyl-1-butanol	169.3 ± 26.87	159.42 ± 19.53 b	163.48 ± 15.28	191.79 ± 5.08	183.72 ± 4.68 a	185.79 ± 8.01
**48**	123-51-3	3-Methyl-1-butanol	285.38 ± 38.23	272.54 ± 23.04 b	280.04 ± 12.50	315.38 ± 8.22	307.74 ± 13.02 a	309.07 ± 17.50
**49**	71-41-0	1-Pentanol	0.14 ± 0.02 a	0.13 ± 0.03 a	0.15 ± 0.02 a	0.08 ± 0.02 b	0.06 ± 0.00 b	0.07 ± 0.01 b
**50**	97-95-0	2-Ethyl-1-butanol	1.11 ± 0.08	1.08 ± 0.02	2.83 ± 0.08	nd	nd	nd
**51**	626-89-1	4-Methyl-1-pentanol	0.54 ± 0.06	0.55 ± 0.09 a	0.50 ± 0.07	0.51 ± 0.05	0.43 ± 0.02 b	0.46 ± 0.02
**52**	543-49-7	2-Heptanol	0.55 ± 0.05	0.51 ± 0.14	0.97 ± 0.96 a	0.28 ± 0.11	0.23 ± 0.03	0.25 ± 0.03 b
**53**	42072-39-9	(*S*)-(+)-3-Methyl-1-pentanol	2.13 ± 0.13	2.33 ± 0.62 a	1.92 ± 0.25	1.86 ± 0.13	1.63 ± 0.03 b	1.57 ± 0.11
**54**	584-02-1	3-Pentanol	0.09 ± 0.04Aa	0.06 ± 0.03 B	0.11 ± 0.05 Aa	0.05 ± 0.01 b	0.04 ± 0.01	0.03 ± 0.01 b
**55**	928-97-2	*E*-3-Hexen-1-ol	0.18 ± 0.05 a	0.15 ± 0.00 a	0.15 ± 0.03	0.12 ± 0.03 b	0.11 ± 0.01 b	0.11 ± 0.01
**56**	111-35-3	3-Ethoxy-1-propanol	0.06 ± 0.03 a	0.05 ± 0.02 a	0.07 ± 0.02 a	0.02 ± 0.00 b	0.01 ± 0.00 b	0.01 ± 0.00 b
**57**	928-96-1	*Z*-3-Hexen-1-ol	0.18 ± 0.04	0.24 ± 0.09	0.19 ± 0.04	0.17 ± 0.04	0.25 ± 0.10	0.24 ± 0.06
**58**	589-98-0	3-Octanol	0.15 ± 0.02 a	0.15 ± 0.03 a	0.16 ± 0.03 a	0.12 ± 0.02 b	0.09 ± 0.00 b	0.10 ± 0.01 b
**59**	928-95-0	*E*-2-Hexen-1-ol	0.24 ± 0.02 AB	0.58 ± 0.44 Aa	0.14 ± 0.01 B	0.04 ± 0.00	0.06 ± 0.01 b	0.16 ± 0.01
**60**	13231-81-7	3-Methyl-1-hexanol	0.04 ± 0.01	0.04 ± 0.01	0.04 ± 0.01	0.05 ± 0.00	0.05 ± 0.00	0.05 ± 0.00
**61**	928-94-9	*Z*-2-Hexen-1-ol	0.05 ± 0.02 a	0.04 ± 0.01 a	0.04 ± 0.01 a	0.03 ± 0.01 b	0.02 ± 0.00 b	0.03 ± 0.00 b
**62**	123-96-6	2-Octanol	0.08 ± 0.02 a	0.07 ± 0.02 a	0.08 ± 0.03 a	0.04 ±0.01 b	0.03 ± 0.00 b	0.03 ± 0.00 b
**63**	3391-86-4	1-Octen-3-ol	0.77 ± 0.20	0.63 ± 0.12	0.80 ± 0.27	0.84 ± 0.27	0.55 ± 0.10	0.62 ± 0.07
**64**	111-70-6	1-Heptanol	2.37 ± 0.50 Aa	1.57 ± 0.49 B	2.01 ± 0.60 ABa	1.4 ± 0.17 b	0.99 ± 0.15	1.11 ± 0.24 b
**65**	1569-60-4	6-Methyl-5-hepten-2-ol	0.02 ± 0.0	0.04 ± 0.01	0.03 ± 0.01	0.03 ± 0.01	0.02 ± 0.00	0.02 ± 0.00
**66**	104-76-7	2-Ethyl-1-hexanol	1.97 ± 0.21 Ba	2.39 ± 0.52 Aa	2.11 ± 0.27 ABa	1.54 ± 0.14 b	1.63 ± 0.06 b	1.58 ± 0.15 b
**67**	38514-13-5	3-Ethyl-4-methyl-1-pentanol	2.65 ± 0.41 a	3.14 ± 1.02 a	3.42 ± 0.81 a	0.48 ± 0.02 b	0.49 ± 0.21 b	0.41 ± 0.10 b
**68**	628-99-9	2-Nonanol	0.58 ± 0.03	0.53 ± 0.02	0.64 ± 0.25 a	0.48 ± 0.030	0.48 ± 0.00	0.46 ± 0.01 b
**69**	111-87-5	1-Octanol	3.74 ± 1.46	2.99 ± 1.41	3.00 ± 1.31	2.94 ± 0.48	2.53 ± 0.36	2.5 ± 0.51
**70**	1653-40-3	6-Methyl-1-heptanol	0.17 ± 0.02	0.27 ± 0.10	0.19 ± 0.02	0.19 ± 0.01	0.19 ± 0.02	0.2 ± 0.02
**71**	18409-17-1	*E*-2-Octen-1-ol	0.17 ± 0.05	0.19 ± 0.07	0.16 ± 0.03	0.16 ± 0.01	0.13 ± 0.01	0.16 ± 0.01
**72**	110453-78-6	(*S*)-(+)-6-Methyl-1-octanol	0.13 ± 0.01 B	0.24 ± 0.1 A	0.15 ± 0.0 AB	0.17 ± 0.0	0.17 ± 0.01	0.19 ± 0.02
**73**	3054-92-0	2,3,4-trimethyl-3-pentanol	0.09 ± 0.01	0.09 ± 0.01	0.13 ± 0.09	0.17 ± 0.05	0.13 ± 0.02	0.16 ± 0.03
**74**	143-08-8	1-Nonanol	0.93 ± 0.30	0.70 ± 0.20	0.71 ± 0.22	0.96 ± 0.14	0.74 ± 0.08	0.82 ± 0.03
**75**	10340-23-5	*Z*-3-Nonen-1-ol	0.09 ± 0.02	0.07 ± 0.0 a	0.1 ± 0.01 a	0.07 ± 0.02	0.04 ± 0.01 b	0.07 ± 0.02 b
**76**	35854-86-5	6-*cis*-Nonenol	0.22 ± 0.10	0.11 ± 0.03	0.16 ± 0.04	0.22 ± 0.10	0.13 ± 0.05	0.22 ± 0.01
**77**	112-72-1	1-Tetradecanol	0.41 ± 0.09	0.4 ± 0.06	0.39 ± 0.13	0.47 ± 0.10	0.48 ± 0.12	0.47 ± 0.11
**78**	36653-82-4	1-Hexadecanol	0.12 ± 0.04	0.12 ± 0.06	0.1 ± 0.00	0.11 ± 0.08	0.07 ± 0.02	0.10 ± 0.04

These data are from the means of four values ± standard deviation (SD). Different upper case letters in each row indicate statistically significant differences (*p* < 0.05) between treatments in the same year, as determined with Fisher’s least significant different (LSD) test. Different lower case letters in each row indicate statistically significant differences (*p* < 0.05) between years for the same treatment, as determined with Fisher’s test. nd = not detected.

**Table 5:** Effects of defoliation treatments and vintage on concentrations of aromatic esters and aromatic alcohols (μg/L). (The quantification was conducted by using a linear standard calibration with 2-phenylethanol.) C = control; FL = removal of six leaves before flowering; VER = removal of six leaves at the end of veraison.

Code	CAS	Name	C2017	FL2017	VER2017	C2018	FL2018	VER2018
**79**	470-82-6	1,8-Cineole	0.57 ± 0.05	0.37 ± 0.01	0.34 ± 0.02	0.66 ± 0.05	1.05 ± 0.47	1.60 ± 0.16
**80**	100-42-5	Ethenyl-benzene	15.49 ± 9.74	19.83 ± 1.33	24.14 ± 1.15 a	13.45 ± 4.75	11.30 ± 1.14	10.32 ± 1.14 b
**81**	93-89-0	Benzoic acid ethyl ester	4.83 ± 1.06	6.14 ± 0.07 a	4.13 ± 0.07	3.14 ± 1.15	2.44 ± 0.20 b	2.47 ± 0.20
**82**	98-00-0	2-Furanmethanol	6.77 ± 3.8	5.56 ± 1.34	5.68 ± 1.02	4.40 ± 1.82	4.08 ± 1.14	3.90 ± 1.05
**83**	101-97-3	Benzeneacetic acid ethyl ester	24.03 ± 15.27	26.18 ± 12.47	16.37 ± 6.33	12.08 ± 1.90	10.11 ± 0.89	8.49 ± 1.28
**84**	103-45-7	Phenylethyl acetate	265.03 ± 87.21 b	235.83 68.88 b	211.59 ± 117.23 b	1021.59 ± 458.85 a	1026.41 ± 358.42 a	697.21 ± 329.40 a
**85**	100-51-6	Benzyl alcohol	11.52 ± 3.92 a	12.02 ± 5.05 a	14.92 ± 4.16 a	5.74 ± 2.06 b	3.33 ± 0.43 b	3.88 ± 0.80 b
**86**	2021-28-5	Benzenepropanoic acid ethyl ester	5.47 ± 0.75	5.16 ± 1.49	17.35 ± 24.51	3.99 ± 0.16	4.13 ± 0.96	3.50 ± 0.36
**87**	122-72-5	Benzenepropanol acetate	1.40 ± 0.52	0.83 ± 0.07	1.71 ± 0.91	0.42 ± 0.08	0.35 ± 0.28	0.31 ± 0.02
**88**	103-36-6	3-Phenyl-2-propenoic acid ethyl ester	21.87 ± 6.03	20.48 ± 12.01 b	26.25 ± 9.03	28.48 ± 9.09 B	48.31 ± 15.28 Aa	32.27 ± 6.16 B
**89**	122-99-6	2-Phenoxyethanol	7.15 ± 4.60	7.87 ± 6.06	5.01 ± 3.34	9.11 ± 4.18	11.74 ± 7.70	8.27 ± 2.92
**90**	97-53-0	Eugenol	3.95 ± 1.73	2.47 ± 0.21	3.05 ± 0.48	3.05 ± 0.22 A	1.13 ± 0.03 B	1.43 ± 0.25 AB
**91**	7786-61-0	*p*-Vinylguaiacol	53.57 ± 1.72	93.52 ± 6.44 a	67.14 ± 5.99 a	26.24 ± 12.95	16.97 ± 7.88 b	23.28 ± 6.75 b
**92**	6259-76-3	2-Hydroxy-benzoic acid-hexyl ester	9.44 ± 1.49	9.15 ± 1.45	9.37 ± 1.67	9.25 ± 1.67	9.21 ± 1.67	9.5 ± 0.90
**93**	91-10-1	2,6-Dimethoxy-phenol	9.74 ± 1.73	10.00 ± 2.08	10.45 ± 1.29 a	6.93 ± 1.15	8.07 ± 1.72	7.68 ± 1.85 b
**94**	15399-05-0	Ethyl-2-hydroxy-3-phenylpropanoate	74.94 ± 7.12	110.33 ± 49.62	104.56 ± 67.66	101.3 ± 29.03	78.32 ± 13.54	85.24 ± 11.3
**95**	118-60-5	Benzoic acid, 2-hydroxy-,2-ethylhexyl ester	67.84 ± 5.78	43.33 ± 3.97	66.82 ± 6.36	21.51 ± 1.93	27.69 ± 1.26	24.71 ± 6.91
**96**	496-16-2	2,3-Dihydro-benzofuran	13.92 ± 2.28 b	17.76 ± 4.30 b	16.11 ± 0.88 b	25.27 4.26 a	25.54 ± 4.05 a	25.68 ± 3.29 a
**97**	617-05-0	4-Hydroxy-3-methoxy-benzoic acid ethyl ester	149.76 ± 40.76 a	175.15 ± 73.62 a	151.43 ± 9.36 a	45.38 ± 12.17 b	65.12 ± 18.72 b	42.78 ± 23.5 b

These data are from the means of four values ± standard deviation (SD). Different upper case letters in each row indicate statistically significant differences (*p* < 0.05) between treatments in the same year, as determined with Fisher’s least significant different (LSD) test. Different lower case letters in each row indicate statistically significant differences (*p* < 0.05) between years for the same treatment, as determined with Fisher’s test.

**Table 6:** Effects of defoliation treatments and vintage on concentrations of ketones (μg/L). (The quantification was conducted by using *β*-damascenone.) C = control; FL = removal of six leaves before flowering; VER = removal of six leaves at the end of veraison.

Code	CAS	Name	C2017	FL2017	VER2017	C2018	FL2018	VER2018
**98**	67-64-1	2-Propanone	6.29 ± 2.63	6.12 ± 3.49	5.92 ± 2.32	4.73 ± 1.21	4.58 ± 1.20	4.98 ± 0.82
**99**	110-43-0	2-Heptanone	24.72 ± 6.83	27.14 ± 3.25	27.78 ± 1.88	4.65 ± 0.26	4.63 ± 0.94	5.75 ± 0.21
**100**	513-86-0	3-Hydroxy-2-butanone	10.41 ± 0.96 a	6.00 ± 0.79	7.36 ± 0.73	0.85 ± 0.04 b	0.73 ± 0.05	0.84 ± 0.00
**101**	116-09-6	1-Hydroxy-2-propanone	11.95 ± 4.95 a	9.79 ± 2.16	11.51 ± 1.62	8.12 ± 1.56 b	7.38 ± 1.14	8.02 ± 1.73
**102**	4485-09-0	4-Nonanone	2.29 ± 0.24	4.75 ± 0.89	3.04 ± 0.22	nd	nd	nd
**103**	110-93-0	6-Methyl-5-hepten-2-one	9.18 ± 3.03	6.18 ± 2.24	6.92 ± 1.07	6.02 ± 0.45	6.47 ± 0.42	6.88 ± 0.32
**104**	821-55-6	2-Nonanone	18.85 ± 5.83	22.73 ± 2.23 a	20.43 ± 1.46	6.19 ± 1.50	6.13 ± 1.50 b	6.22 ± 0.79
**105**	39178-69-3	3-Butyl-cyclohexanone	0.77 ± 0.07	0.52 ± 0.03	0.82 ± 0.02	nd	nd	nd
**106**	928-80-3	3-Decanone	20.03 ± 0.47	20.21 ± 0.31	20.45 ± 0.37	20.42 ± 0.13	20.42 ± 0.10	20.17 ± 0.06
**107**	13679-85-1	Dihydro-2-methyl-3(2*H*)-thiophenone	2.77 ± 0.03 b	1.57 ± 0.87 b	3.73 ± 0.21 b	16.39 ± 3.24 Aa	10.92 ± 2.15 Ba	13.57 ± 2.10 ABa
**108**	96-48-0	Dihydro-2(3*H*)-furanone	15.31 ± 1.19 b	17.08 ± 2.05 b	15.28 ± 1.62 b	24.53 ± 4.36 a	28.37 ± 2.82 a	25.92 ± 3.38 a
**109**	98-86-2	Acetophenone	32.4 ± 1.83 a	40.91 ± 7.35 a	32.73 ± 2.95 a	7.37 ± 1.15 b	7.28 ± 1.16 b	6.64 ± 0.95 b
**110**	6175-49-1	2-Dodecanone	14.4 ± 3.14	13.89 ± 1.87	13.86 ± 2.20	12.58 ± 2.26	12.62 ± 3.16	12.79 ± 3.88
**111**	22122-36-7	3-Methyl-2(5*H*)-furanone	7.54 ± 0.56	12.36 ± 2.31	7.98 ± 0.96	5.2 ± 0.74	2.69 ± 1.26	6.17 ± 0.74
**112**	3796-70-1	Geranylacetone	6.88 ± 1.15	5.86 ± 1.04	6.28 ± 1.57	7.58 ± 3.98	7.81 ± 3.44	7.73 ± 3.14
**113**	104-61-0	2(3*H*)-Furanone, dihydro-5-pentyl-	299.13 ± 14.15	239.37 ± 13.5	189.23 ± 35.66	180.56 ± 45.74	131.24 ± 11.91	157.51 ± 17.18
**114**	706-14-9	*γ*-Decanolactone	49.06 ± 8.4 A	39.97 ± 7.68 Bb	37.61 ± 1.50 Bb	51.94 ± 1.17	48.67 ± 3.49 a	50.02 ± 3.48 a
**115**	705-86-2	*δ*-Decalactone	20.91 ± 1.22	22.44 ± 6.05 a	20.28 ± 0.71	17.39 ± 1.73	17.25 ± 1.42 b	16.94 ± 1.24
**116**	710-04-3	*δ*-Undecalactone	19.5 ± 2.21 b	17.58 ± 1.00 b	17.92 ± 0.81 b	23.76 ± 1.38 a	23.15 ± 2.55 a	24.49 ± 4.00 a
**117**	713-95-1	*δ*-Dodecalactone	18.32 ± 6.85	18.69 ± 8.73	15.64 ± 2.47	16.18 ± 1.52	16.11 ± 0.94	15.71 ± 1.30

These data are from the means of four values ± standard deviation (SD). Different upper case letters in each row indicate statistically significant differences (*p* < 0.05) between treatments in the same year, as determined with Fisher’s least significant different (LSD) test. Different lower case letters in each row indicate statistically significant differences (*p* < 0.05) between years for the same treatment, as determined with Fisher’s test. nd = not detected.

**Table 7:** Effects of defoliation treatments and vintage on concentrations of the aldehydes, monoterpenoles, and monoterpenes (μg/L). (The quantification was conducted in the same manner as Table 6). C = control; FL = removal of six leaves before flowering; VER = removal of six leaves at the end of veraison.

Code	CAS	Name	C2017	FL2017	VER2017	C2018	FL2018	VER2018
**118**	78-84-2	2-Methyl-propanal	0.12 ± 0.05 a	0.1 ± 0.02 a	0.1 ± 0.01	0.02 ± 0.00 b	0.01 ± 0.00 b	0.06 ± 0.02
**119**	123-72-8	Butanal	0.12 ± 0.01	0.01 ± 0.00	0.03 ± 0.00	nd	nd	nd
**120**	590-86-3	3-Methyl-butanal	0.62 ± 0.02	0.7 ± 0.35 a	0.4 4 ± 0.02	0.33 ± 0.02	0.17 ± 0.01 b	0.4 ± 0.10
**121**	4170-30-3	2-Butenal	0.19 ± 0.02	nd	nd	nd	nd	nd
**122**	66-25-1	Hexanal	0.08 ± 0.03	0.1 ± 0.04	0.07 ± 0.02	0.04 ± 0.0	0.05 ± 0.01	0.06 ± 0.01
**123**	111-71-7	Heptanal	0.06 ± 0.02	0.07 ± 0.03	0.06 ± 0.02	0.07 ± 0.02	0.06 ± 0.02	0.06 ± 0.02
**124**	124-13-0	Octanal	0.17 ± 0.05	0.18 ± 0.01	0.17 ± 0.02	0.17 ± 0.01	0.15 ± 0.08	0.17 ± 0.04
**125**	124-19-6	Nonanal	0.40 ± 0.10	0.41 ± 0.10	0.39 ± 0.01	0.40 ± 0.02	0.42 ± 0.02	0.38 ± 0.02
**126**	98-01-1	Furfural	0.43 ± 0.02	0.34 ± 0.05	0.35 ± 0.04	0.36 ± 0.40	0.35 ± 0.10	0.31 ± 0.05
**127**	112-31-2	Decanal	0.30 ± 0.09	0.31 ± 0.08	0.30 ± 0.07	0.34 ± 0.17	0.37 ± 0.19	0.34 ± 0.13
**128**	100-52-7	Benzaldehyde	0.46 ± 0.05	0.23 ± 0.09	0.23 ± 0.04	0.20 ± 0.05	0.33 ± 0.01	0.25 ± 0.09
**129**	18829-56-6	*E*-2-Nonenal	0.01 ± 0.00 b	0.02 ± 0.00 b	0.01 ± 0.00 b	0.03 ± 0.00 a	0.04 ± 0.00 a	0.03 ± 0.00 a
**130**	4411-89-6	2-Phenyl-2-butenal	0.11 ± 0.02	nd	nd	nd	nd	nd
**131**	18479-58-8	2,6-Dimethyl-7-octen-2-ol	0.42 ± 0.03	0.37 ± 0.09	0.47 ± 0.02	0.47 ± 0.06	0.31 ± 0.03	0.31 ± 0.01
**132**	98-55-5	*α*-Terpineol	0.22 ± 0.02	0.36 ± 0.02 a	0.27 ± 0.09	0.17 ± 0.06	0.15 ± 0.01 b	0.14 ± 0.02
**133**	106-22-9	*β*-Citronellol	2.75 ± 0.39	3.21 ± 1.81	2.55 ± 0.70	2.71 ± 0.46	2.54 ± 0.21	2.66 ± 0.11
**134**	127-91-3	*β*-Pinene	0.08 ± 0.02	0.11 ± 0.03 a	0.11 ± 0.01 a	0.06 ± 0.02	0.07 ± 0.01 b	0.07 ± 0.03 b

These data are from the means of four values ± standard deviation (SD). Different lower case letters in each row indicate statistically significant differences (*p* < 0.05) between years for the same treatment, as determined with Fisher’s test. nd = not detected.

## Data Availability

Not available.

## Sample Availability

Samples of the compounds are not available from the authors.
